# Edge-Enabled Hybrid Encryption Framework for Secure Health Information Exchange in IoT-Based Smart Healthcare Systems

**DOI:** 10.3390/s25247583

**Published:** 2025-12-14

**Authors:** Norjihan Abdul Ghani, Bintang Annisa Bagustari, Muneer Ahmad, Herman Tolle, Diva Kurnianingtyas

**Affiliations:** 1 Department of Information Systems, Faculty of Computer Science and Information Technology, Universiti Malaya, Kuala Lumpur 50603, Malaysia; bintang.bagustari@um.edu.my; 2 Faculty of Computer Science, Universitas Brawijaya, Jl. Veteran No. 10-11, Malang 65145, Jawa Timur, Indonesia; emang@ub.ac.id (H.T.); divaku@ub.ac.id (D.K.); 3 Department of Computer Science, University of Roehampton, London SW15 5PH, UK

**Keywords:** IoT-based smart healthcare, edge-enabled encryption, health information exchange, hybrid algorithms

## Abstract

The integration of the Internet of Things (IoT) and edge computing is transforming healthcare by enabling real-time acquisition, processing, and exchange of sensitive patient data close to the data source. However, the distributed nature of IoT-enabled smart healthcare systems exposes them to severe security and privacy risks during health information exchange (HIE). This study proposes an edge-enabled hybrid encryption framework that combines elliptic curve cryptography (ECC), HMAC-SHA256, and the Advanced Encryption Standard (AES) to ensure data confidentiality, integrity, and efficient computation in healthcare communication networks. The proposed model minimizes latency and reduces cloud dependency by executing encryption and verification at the network edge. It provides the first systematic comparison of hybrid encryption configurations for edge-based HIE, evaluating CPU usage, memory consumption, and scalability across varying data volumes. Experimental results demonstrate that the ECC + HMAC-SHA256 + AES configuration achieves high encryption efficiency and strong resistance to attacks while maintaining lightweight processing suitable for edge devices. This approach provides a scalable and secure solution for protecting sensitive health data in next-generation IoT-enabled smart healthcare systems.

## 1. Introduction

Smart city-based IoT such as smart healthcare enables the collection and transmission of vast amounts of health data and allows people and things to be connected anywhere, any time, and to anyone. It has led to the development of IoT-based smart healthcare systems, which enable real-time data collection and sharing of patient information. According to Butt et al. [1], there are four steps to follow for the development of a smart healthcare system: (i) complete knowledge to abstract information from intelligent things connected with the network, (ii) maintenance and transmission of data, (iii) intelligent processing and decision making by the system, and (iv) securing from vulnerabilities.

Ghamari et al. emphasized the importance of smart healthcare systems in healthcare organizations [2]. Meanwhile, Manogaran et al. acknowledged the rapid growth of IoT technology, which is expected to bring various healthcare applications [3]. However, there are still major concerns related to security and privacy, particularly in the context of health information exchange (HIE).

HIEs can improve healthcare delivery by providing the ability to securely share healthcare information electronically across multiple healthcare organizations reliably and securely [4]. HIE allows the sharing of healthcare information electronically among various organizations, while ensuring reliability and security in the edge computing environment. The main security issue in HIE is the protection of electronic patient records during transmission [5]. According to [6], patient data becomes more vulnerable to unauthorized access without the implementation of any proper security measures, such as encryption. Furthermore, the absence of suitable security measures may pose any potential risks not only to patient privacy but also to the integrity of the healthcare system and data itself.

Several studies have highlighted the importance of the security aspect in IoT-based smart healthcare, especially when HIE is involved. The absence of security measures may lead to patient data leakage or unauthorized data access, which compromises confidentiality [5]. A CIA triad is crucial for secure HIE, which shows the need to prevent any inappropriate data access, protect data from unauthorized alteration, and ensure reliable access to information [7].

Most of the traditional approaches for HIE emphasize processing time, but these approaches do not consider the resource constraints and scalability requirements which are important in smart healthcare environments. Focusing on safeguarding healthcare data, specifically personally identifiable information of patients and their health records, this study concentrates on two of the main goals: first, to improve the quality of life of citizens with smart healthcare, and second, to guarantee their privacy.

This study makes several key contributions to the field of secure HIE:

1. Comparative analysis: A comparison of the proposed techniques and the performance metrics used. A detailed comparison of different hybrid encryption approaches is provided, providing valuable insights related to the performance metrics used. Unlike prior studies that focus narrowly on encryption/decryption time, this work evaluates CPU usage, memory consumption, and scalability across varying file sizes—providing a holistic view of algorithm suitability for dynamic healthcare data environments.
2. Edge-enabled with a multi-layer approach: a novel edge-enabled multi-layer encryption framework that combines ECC for secure key exchange, HMAC-SHA256 for authentication, and multiple symmetric algorithms for confidentiality. By executing key exchange, authentication, and encryption at the network edge, the system ensures real-time responsiveness and lightweight processing—critical for smart healthcare systems operating with limited computational resources. This edge-enabled encryption proposes a dynamic mechanism that implement the strategy based on file size and resource constraints.
3. Holistic performance-centric evaluation: An extensive evaluation is used, with computational time, CPU usage, memory usage, and scalability aspects, providing a significant evaluation for IoT-based smart healthcare environments. While most of the previous studies focus only on encryption/decryption times, this research provides a more holistic performance analysis.
4. Scalability assessment: The assessment involves evaluating the algorithm’s performance using various file sizes. This approach demonstrates the consistent performance even with different file sizes, which provides a significant insight into how different cryptographic solutions scale in dynamic IoT-based smart healthcare data environments.
5. Seamless integration into HIE workflows: The architecture aligns with real-world HIE scenarios, enabling secure data exchange between healthcare entities (e.g., Hospital A to Hospital B), using ECC for key agreement, HMAC-SHA256 for integrity verification, and symmetric encryption for confidentiality. This layered approach supports compliance with the CIA triad and enhances interoperability across heterogeneous platforms.

## 2. Related Works

IoT-based smart healthcare systems facilitate real-time monitoring, early anomaly detection, and personalized treatment plans, ultimately improving patient care and outcomes. The seamless integration of IoT in healthcare has revolutionized the way medical data is collected, analyzed, and utilized, paving the way for more efficient, accurate, and patient-centric healthcare delivery [5].

### 2.1. Health Information Exchange Security Requirements

HIE, which is a truly interoperable connected health system, enables data flows with both one-to-one and one-to-many connections, leading to the exchange of information among multiple interfaces which require systems to cooperate with one another. However, security issues for electronic patient records in transit hinder the usage of IoT in smart healthcare systems. Among these issues, the confidentiality and entity authentication of electronic patient records are major concerns [5]. Furthermore, where HIE requires more sensitive data to be collected and shared among various platforms, protection against unauthorized use/access is essential. Unauthorized access to IoT devices could create a serious risk to patient’s health, as well as to their private information. Maintaining the security and privacy of patients’ health data is the biggest challenge in smart healthcare systems [7].

Another important aspect that requires attention in IoT-based smart healthcare is the lack of interoperability among the heterogeneous platforms, which is identified as a potential vulnerability that could lead to data privacy loss, compliance regulation issues, and backward compatibility with legacy systems. Internal sharing of electronic medical records is the norm [8]. Data leakage may occur throughout the sharing process between multiple healthcare organizations, as a result of human mistakes or flaws in the procedure or technology. Therefore, ensuring that the utmost priority is given to the security of patient data is crucial. Implementing comprehensive protection measures, including data encryption, user authentication, and application security, while adhering to current security standards and validation algorithms, is imperative [9].

The expansions of HIEs may make it possible for their medical records to be accessible in a wider variety of healthcare settings. Thus, their concerns may grow if numerous entities and organizations can access and examine the clinical data of the patients. The establishment of IoT-based smart healthcare systems also relies on the sensing devices that are usually deployed in the open environment where numerous security risks exist. To ensure the security of sensitive data, a system should only restrict access to authorized users. Consequently, safeguarding the data against unauthorized access becomes crucial [10]. Therefore, enhancing the quality of healthcare and improving ease of access to health records while maintaining the security and privacy of the data is crucial and challenging for healthcare organizations globally.

### 2.2. Cryptographic Techniques for Secure Smart Healthcare Systems

The reviewed literature focuses on the application of security techniques in the healthcare context. The literature review was carried out not only to identify the importance of performance optimization but also to influence the main goal of the study.

Existing studies have explored various cryptographic methods to secure HIE. Khan et al. (2020) proposed a secure framework for authentication and encryption of IoT-based medical sensors [9]. An integration of data substitution–Caesar cipher and improved elliptical curve cryptography (IECC) is presented in this research and was simulated using Java and NS3; however, the simulation does not accurately represent the real-world performance of smart healthcare.

Popoola et al. (2024) proposed an optimized hybrid encryption framework which combined ECC-256R1 and AES-128 in EAX mode [11]. This solution is implemented in a smart home healthcare environment, capable in mitigating common cybersecurity threats, such as man-in-the-middle and replay attacks. This research also highlights the need of lightweight and efficient cryptographic techniques for securing sensitive health data in IoT applications.

Meanwhile, Lahraoui et al. (2024) introduced a hash-based technique to securely embed messages into elliptic curve points, to enhance elliptic curve cryptography (ECC) during the HIE [12]. Random parameters were used and secret points were shared from the elliptic curve of the Diffie–Hellman protocol to improve security against various attack models. The findings show that the proposed scheme maintained the linear complexity and ensured the integrity of the message via securely attached tags.

Nagarajan et al. (2021) utilized the RES-256 Algorithm with a more secure setup from any potential known attacks such as denial of service, attack blocking, or sensor attacks, in order to strengthen the safety of the patient data [13]. Meanwhile, Qiu et al. (2020) used a proposed AES Encryption, which was able to provide high-level protection during data sharing, improve data integrity, and perform more efficiently with a smartphone platform [14]. Therefore, this approach is not suitable to be implemented in large-scale data sharing scenarios, due to high computational and storage overhead, which raise conflicts with the scenario of smart healthcare environments.

Kaliyaperumal and Sammy (2022) introduced Attribute-Based Encryption (ABE) with the aim of addressing security challenges such as scalability, access flexibility, and account revocation, specifically in medical applications [10]. In the proposed technique, an assumption is made where the third-party servers are considered secure and reliable, which is not the usual protocol. Xiong et al. (2020) proposed Ciphertext-Policy Attribute-based encryption, which provides powerful security with less computational overhead than the existing schemes, but may face challenges if sensitive medical information is being collected and shared [15]. The Blowfish Algorithm proposed by [16], with increased efficiency and key expansion, is thought to be effective and secure. However, it does not address interoperability with any current security mechanisms or protocols in the healthcare sector.

Table 1 shows that the existing studies tend to prioritize encryption and decryption time as the main performance indicators, but a more comprehensive assessment of cryptographic solutions’ suitability for HIE should consider a wider range of factors. This includes evaluating the algorithms’ ability to efficiently manage memory resources and maintain consistent performance as data volumes increase. Smart healthcare environments often involve large amounts of sensitive patient data, and the ability of security measures to efficiently handle growing data quantities is crucial. Thus, the existing research has notable limitations. There is a lack of comprehensive analysis regarding the scalability and memory utilization of these cryptographic techniques. Most studies primarily focus on encryption and decryption efficiency, without adequately addressing how the algorithms perform in terms of memory usage and scalability when dealing with varying file sizes and data volumes. These gaps underscore the need for holistic, performance-aware encryption strategies suitable for dynamic healthcare environments.

Table 2 shows the comparative summary of existing works and our proposed framework. This work advances the state of the art by bridging cryptographic rigor with practical deployment needs in smart healthcare. It offers a scalable, secure, and performance-aware solution for protecting sensitive patient data in next-generation HIE systems.

### 2.3. Edge-Enabled Encryption

Edge-enabled hybrid encryption framework distinguishes itself from some prior works through its comprehensive approach to health information exchange across IoT-based smart healthcare ecosystems. While Gowthamani et al. focus on edge cryptographic strategies [18], Singh & Chatterjee on health monitoring security [19], and Parah et al. on authentication mechanisms [20], our work uniquely integrates a hybrid encryption architecture that strategically combines symmetric and asymmetric cryptographic methods across multiple system layers (device, edge, cloud). The key distinction lies in our framework’s emphasis on secure health information exchange between healthcare entities rather than just internal data protection, addressing security and performance optimization challenges. By distributing encryption tasks intelligently based on computational capabilities, our hybrid approach provides a more comprehensive solution for securing data throughout the entire healthcare information exchange lifecycle, from IoT sensors through edge processing to inter-organizational data sharing, which previous works do not address as holistically.

## 3. Proposed Framework

The following section explains the hybrid encryption architecture designed specifically for secure information exchange in IoT-based smart healthcare environments. We outline the key components and structure of the proposed edge-enabled hybrid encryption architecture, highlighting its tailored approach for IoT-based smart healthcare systems.

### 3.1. Information Exchange in IoT-Based Smart Healthcare

In normal data sharing between hospital, patients’ data from Hospital A should be able to be shared to Hospital B whenever requested. It contains patients’ medical records or general patient information that were stored in Hospital A’s repository, which is then exchanged across the HIE network to Hospital B’s repository upon request. During this request, an edge-enabled hybrid algorithm, which consists of a secure key exchange algorithm, hashing function, and symmetric encryption algorithm, can be applied when Hospital A is trying to send the data to Hospital B to ensure confidentiality, whereby encrypted messages cannot be viewed by attackers who do not own the decryption keys.

### 3.2. Overview of the Proposed Architecture

**Figure 1:**
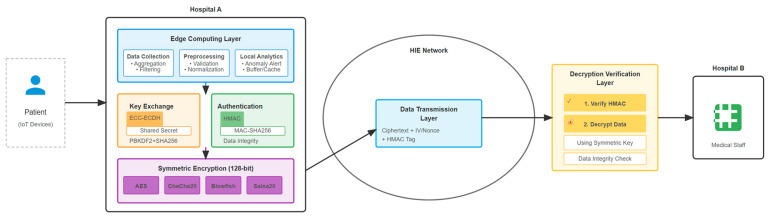
Proposed architecture.

To address the limitations identified in the existing approaches discussed in Section 2, this study proposed an edge-enabled hybrid encryption approach that integrates multiple security layers tailored for secure information exchange in IoT-based smart healthcare environments. Figure 1 shows how the proposed architecture fits into the IoT-based smart healthcare environment, which consists of four layers. The patients’ data is being collected and stored in Hospital A’s repository; meanwhile, Hospital B, who requested the data, send the request via the HIE network within an edge computing environment. This architecture offers superior performance metrics while maintaining the utmost security of patients’ medical records during information exchange. The architecture combines the following:

○ Elliptic curve cryptography (ECC) for secure key exchange;
○ Hash-based message authentication code (HMAC) with SHA256 for data integrity and authentication;
○ Symmetric encryption algorithms (AES, Salsa20, Blowfish, ChaCha20) for efficient data encryption.

This approach is designed to ensure compliance with the CIA triad while maintaining low computational overhead in an edge computing environment.

### 3.3. Key Components of the Architecture

The proposed edge-enabled hybrid encryption algorithm combines ECC and HMAC with a symmetric cryptographic algorithm in order to provide a secure transmission between entities (healthcare organizations) by securely exchanging the key that also reduces computational load [16], adds an extra layer of security by adding a hash function in order to verify the data’s authenticity and detect tampering during transmission [21], and provides confidentiality in the data using robust symmetric encryptions [22].

ECC is one of the approaches to public key encryption techniques that is based on the algebraic structure of elliptic curves over finite fields. Since this paper focuses on security and efficiency, ECC is adopted as it is reported to have a high level of security compared to other traditional algorithms such as RSA, making it more suitable for resource-constrained environments like smart healthcare [14,23]. HMAC is used to verify the accuracy of data that is stored or transferred across an unreliable channel [21]. HMAC combines a hash function with a secret key to generate a code that can be used to verify the authenticity and integrity of the data. To elaborate, the hash function uses SHA256 or SHA3 combined with the secret key to create the authentication code. Therefore, upon receiving the message and its corresponding HMAC, the recipient could compute the HMAC of the received message by utilizing the same secret key. In the instance that the values correspond, it can be concluded that the message has remained unaltered during transmission. In this study, SHA256 is used as the hash function as it can generate a compressed message that is smaller than the plaintext. ECC is used as the public key for secure key exchange, and HMAC paired with SHA256 is also used.

To address the varied computational capabilities and latency constraints of edge devices in IoT-based smart healthcare systems, this study integrates four symmetric encryption algorithms: AES, ChaCha20, Blowfish, and Salsa20. Each algorithm is chosen for its distinct performance and security attributes. AES (Advanced Encryption Standard) is widely adopted and benefits from hardware acceleration across many platforms, making it a strong and efficient choice for high-throughput environments [21]. ChaCha20, a stream cipher optimized for software implementations, provides robust resistance to timing attacks and performs exceptionally well on resource-constrained devices lacking dedicated cryptographic hardware [24]. Blowfish is recognized for its simplicity and rapid key setup, making it suitable for encrypting short messages with minimal overhead, though it is less favored for large-scale data due to its smaller block size [25]. Salsa20, the predecessor to ChaCha20, offers comparable speed and security, serving as an alternative for scenarios where lightweight stream ciphers are preferred [26]. By incorporating these algorithms into a modular framework, the system facilitates adaptive encryption strategies based on file size, device capabilities, and latency requirements, ensuring both flexibility and resilience in dynamic healthcare data exchange scenarios. These symmetric algorithms are used for data encryption in this proposed method using the cipher key of 128. A 128-bit key is advisable for systems operating in hard real-time. It is indicated that this key size provides a balance between security and performance [21].

To conclude, this edge-enabled hybrid encryption approach combining ECC (elliptic curve cryptography) for secure key exchange, HMAC-SHA256 for data integrity and authentication, and the encryption algorithms for encryption will create a hybrid cryptographic scheme for confidentiality. This layered approach ensures compliance with the CIA triad while maintaining low computational overhead suitable for edge environments.

The encryption process follows these steps:

(a) Key exchange: Hospital A and Hospital B generate ECC key pairs. Using Elliptic Curve Diffie–Hellman (ECDH), a shared secret is derived;
(b) Key derivation: The shared secret is processed via PBKDF2 with SHA256 and a random salt to produce a symmetric key;
(c) Authentication: HMAC-SHA256 is computed over the plaintext to generate a message authentication code (MAC);
(d) Encryption: The plaintext is encrypted using one of the selected symmetric algorithms with a session specific IV or nonce;
(e) Transmission: The ciphertext, IV/nonce, and HMAC tag are transmitted securely;
(f) Verification and decryption: The receiver verifies the HMAC and decrypts the ciphertext using the derived symmetric key.

**Figure 2:**
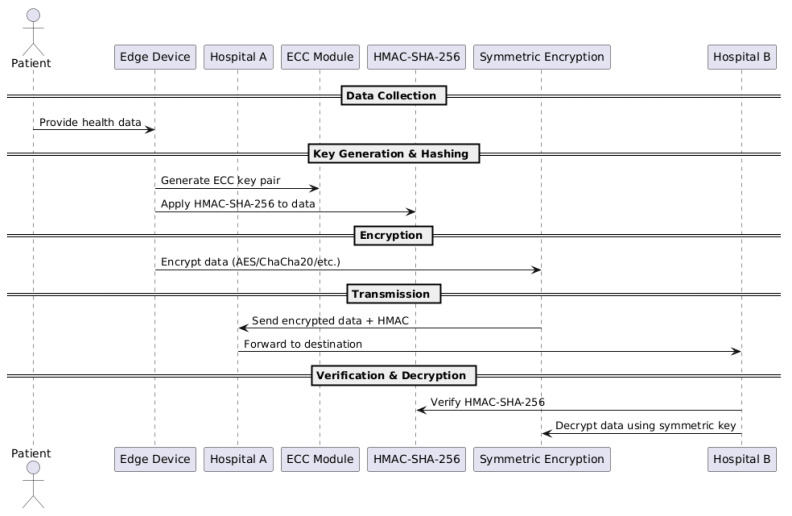
Sequence diagram of the proposed authentication and encryption scheme.

Figure 2 shows the sequence diagram of the Algorithm 1, the proposed edge-enabled hybrid encryption algorithms. The procedure is as follows:

○ ECC is one of the public key encryption techniques for secure key exchange;
○ HMAC with SHA256 is used to generate a cryptographic hash function with a secret key that creates unique code for each message;
○ Symmetric algorithms such as AES, Salsa20, Blowfish, ChaCha20 are used to encrypt the data using the cipher key of 128.

**Algorithm 1**: Proposed authentication and encryption scheme algorithm1. Generate ECC key pair (Hospital A); 2. Receive ECC public key (Hospital B); 3. Derive shared secret via ECDH; 4. Apply PBKDF2 with SHA256 and salt → symmetric key; 5. Generate IV/nonce (algorithm-specific); 6. Compute HMAC-SHA256 over plaintext; 7. Encrypt plaintext using selected symmetric algorithm; 8. Transmit: {ciphertext, IV/nonce, HMAC tag}; 9. Receiver:  a. Derive symmetric key;  b. Verify HMAC;  c. Decrypt ciphertext.

## 4. Proposed Solution and Experiments

As a hybrid approach, the enhancement was studied using datasets, experiment configuration, performance metrics evaluation, scalability, and statistical analysis. To generate the dummy dataset for the experiments, a Python script was developed to create realistic patient information records. The dataset consists of general patient information, including attributes such as Patient ID, Patient Name, Age, Gender, Blood Group, Diagnosis, Medication, and Next Appointment.

### 4.1. Datasets and Setup

Firstly, the script defines the structure of the patient information records by specifying the attributes to be included, such as Patient ID, Patient Name, Age, Gender, Blood Group, Diagnosis, Medication, and Next Appointment. The random values for the patient records are created and then exported as CSV files.

In this healthcare scenario, the dummy dataset is generated, which duplicates real-world patient information, representing realistic data of a smart healthcare environment. Different file sizes are created so that experiments can be conducted for performance evaluation and scalability of the proposed architecture across various data volumes.

### 4.2. Experimental Setup and Requirements

The experiments were employed on a Macbook Air (2017) with a 1.8 GHz Dual-Core Intel Core i5 processor and memory of 8 GB 1600 MHz DDR3, and 512 GB SSD for storage, sufficient capacity for the datasets and generated files. While this configuration does not reflect the constrained computational capacity of embedded or wearable IoT nodes, it was intentionally selected to establish a controlled proof-of-concept environment and ensure consistent benchmarking across hybrid encryption configurations. The MacBook Air supports cross-compilation toolchains (arm-none-eabi, gcc-aarch64) for building binaries targeting ARM Cortex or Jetson platforms.

Python 3.10.9 was chosen as the programming language for implementing the proposed architecture and experiments implementation. The code was written and executed using Visual Studio Code (Version 1.81.0) as the integrated development environment (IDE).

Several cryptographic libraries were utilized to implement the hybrid cryptographic algorithms. These libraries include the following:

- cryptography.hazmat.primitives for basic cryptographic primitives;
- cryptography.hazmat.primitives.ciphers for cipher algorithms;
- cryptography.hazmat.primitives.kdf.pbkdf2 for key derivation functions;
- cryptography.hazmat.primitives.asymmetric import ec for elliptic curve cryptography;
- cryptography.hazmat.backends for cryptographic backends.

Additionally, the psutil library was used to monitor system resources, such as CPU and memory usage, during the execution of the experiments. Using Python and the mentioned cryptographic libraries allowed for efficient implementation of the hybrid cryptographic algorithms, including ECC for key exchange, HMAC-SHA256 for authentication, and symmetric algorithms (AES, Salsa20, Blowfish, ChaCha20) for encryption and decryption. The psutil library facilitated the monitoring and collection of performance metrics, such as CPU and memory usage, during the experiments.

### 4.3. Edge-Enabled Hybrid Algorithms

This study introduced proposed enhanced performance, so 128-bit keys are used for the symmetric algorithms, which is advisable for systems operating in hard real-time [21]. The next section discusses how the approach determines which multi-layer hybrid algorithm is most suitable for a smart healthcare environment.

#### 4.3.1. ECC + HMAC-SHA256 + AES

Algorithm 2 incorporates ECC for secure key exchange with HMAC with SHA256 to hash large amounts of data for quick and efficient authentication and AES for data encryption. The implementation is as follows:
**Algorithm 2:** ECC + HMAC-SHA256 + AES#Generate private ECC key for Hospital A   private_key_a=ec.generate_private_key(ec.SECP224R1(), default_backend()) #Obtain public ECC key of Hospital B: public_key_b = [Received securely from Hospital B] #Generate shared secret using ECDH: shared_secret = private_key_a.exchange(ec.ECDH(), public_key_b) #Generate a 16-byte salt: salt = os.urandom(16) #Derive AES symmetric key from shared secret: key_derivation = PBKDF2HMAC(   algorithm = SHA256,   length = 32,   salt = salt,   iterations = 100,000,   backend = default_backend() ) symmetric_key = key_derivation.derive(shared_secret) #Generate a 16-byte Initialization Vector (IV) for AES: iv = os.urandom(16) #Create AES cipher in CFB mode: cipher = Cipher(algorithms.AES(symmetric_key), modes.CFB(iv), backend=default_backend()) encryptor = cipher.encryptor() #Encrypt plaintext data: plaintext = b”Confidential patient record” ciphertext = encryptor.update(plaintext) + encryptor.finalize() End Algorithm

#### 4.3.2. ECC + HMAC-SHA256 + Chacha20

Algorithm 3 utilizes ECC for key exchange with HMAC with SHA256 to hash large amounts of data quickly and efficiently and Chacha20 for data encryption. The implementation is as follows:
**Algorithm 3:** ECC + HMAC-SHA256 + Chacha20#Generate ECC private key for Hospital A: private_key_a=ec.generate_private_key(ec.SECP224R1(), default_backend()) #Obtain public ECC key of Hospital B: public_key_b = [Hospital B’s ECC public key] #Generate shared secret using ECDH: shared_secret = private_key_a.exchange(ec.ECDH(), public_key_b) #Generate 16-byte salt: salt = os.urandom(16) #Derive a 32-byte symmetric key using PBKDF2HMAC: key_derivation = PBKDF2HMAC(   algorithm = SHA256,   length = 32,       //Required size for ChaCha20   salt = salt,   iterations = 100,000,   backend = default_backend() ) symmetric_key = key_derivation.derive(shared_secret) #Generate a 16-byte nonce for ChaCha20: nonce = os.urandom(16) #Create ChaCha20 cipher: cipher = Cipher(algorithms.ChaCha20(symmetric_key, nonce), mode=None,  backend=default_backend()) #Encrypt data: encryptor = cipher.encryptor() plaintext = b”Confidential patient data from Hospital A” ciphertext = encryptor.update(plaintext) + encryptor.finalize() End Algorithm

#### 4.3.3. ECC + HMAC-SHA256 + Blowfish

Algorithm 4 utilizes ECC for key exchange with HMAC with SHA256 to hash large amounts of data quickly and efficiently and Blowfish for data encryption. The implementation is as follows:
**Algorithm 4:** ECC + HMAC-SHA256 + Blowfish#Generate ECC private key for Hospital A: private_key_a=ec.generate_private_key(ec.SECP224R1(), default_backend()) #Obtain public ECC key of Hospital B: public_key_b = [Hospital B’s ECC public key] #Generate shared secret via ECDH: shared_secret = private_key_a.exchange(ec.ECDH(), public_key_b) #Generate a 16-byte salt: salt = os.urandom(16) #Derive symmetric key using PBKDF2HMAC: key_derivation = PBKDF2HMAC(   algorithm = SHA256,   length = 32,   salt = salt,   iterations = 100,000,   backend = default_backend() ) symmetric_key = key_derivation.derive(shared_secret) #Generate IV for Blowfish (typically 8 bytes): iv = os.urandom(8) #Create Blowfish cipher in CFB mode: cipher = Cipher(algorithms.Blowfish(symmetric_key), modes.CFB(iv), backend=default_backend()) #Encrypt data: encryptor = cipher.encryptor() plaintext = b”Confidential patient data from Hospital A” ciphertext = encryptor.update(plaintext) + encryptor.finalize() End Algorithm

#### 4.3.4. ECC + HMAC-SHA256 + Salsa20

Algorithm 5 utilizes ECC for key exchange with HMAC with SHA256 to hash large amounts of data quickly and efficiently and Salsa20 for data encryption. The implementation is as follows:
**Algorithm 5:** ECC + HMAC-SHA256 + Salsa20#Generate ECC private key for Hospital A: private_key_a=ec.generate_private_key(ec.SECP224R1(), default_backend()) #Obtain public ECC key of Hospital B: public_key_b = [Hospital B’s ECC public key] #Generate shared secret using ECDH: shared_secret = private_key_a.exchange(ec.ECDH(), public_key_b) #Generate a 16-byte salt: salt = os.urandom(16) #Derive symmetric key from shared secret: key_derivation = PBKDF2HMAC(   algorithm = SHA256,   length = 32,       //256-bit key for Salsa20   salt = salt,   iterations = 100,000,   backend = default_backend() ) symmetric_key = key_derivation.derive(shared_secret) #Generate 8-byte nonce for Salsa20 (required): nonce = os.urandom(8) #Create Salsa20 cipher: cipher = Cipher(algorithms.Salsa20(symmetric_key, nonce), mode=None, backend=default_backend()) #Initialize encryptor and encrypt the data: encryptor = cipher.encryptor() plaintext = b”Confidential patient data from Hospital A” ciphertext = encryptor.update(plaintext) + encryptor.finalize() End Algorithm

## 5. Performance and Scalability Evaluation

### 5.1. Performance Evaluation

A comprehensive evaluation of the proposed algorithms has been conducted, focusing on the aspects of performance and scalability. The proposed solution emphasizes performance-centric encryption, particularly in resource-constrained environments common in IoT-based smart healthcare settings. A custom dataset simulating real patient records was generated to benchmark algorithm performance across varying file sizes. The performance metrics used are processing time (encryption/decryption), CPU usage (encryption/decryption), and memory usage (encryption/decryption). Table 3, Table 4 and Table 5 illustrate the performance evaluation for the four edge-enabled hybrid encryption algorithms.

According to [9], file encryption time was measured based on different input file sizes with the same key size of 128. File encryption time indicates the time utilized by the encryption algorithm to create a ciphertext from a plaintext, and file decryption time indicates the time utilized by the decryption algorithm to obtain a plaintext from a ciphertext, as shown in Equations (1) and (2) below.

(1) $$T_{enc}=\frac{S}{R_{enc}}+T_{overhead}$$

(2) $$T_{dec}=\frac{S}{R_{dec}}+T_{overhead}$$

where

$T_{enc}$ = total encryption time (seconds)

$T_{dec}$ = total decryption time (seconds)

S = file size

$R_{enc}$ = Encryption speed

$R_{dec}$ = Decryption speed

$T_{overhead}$ = Overhead time (seconds)

CPU usage (encryption and decryption) measured the percentage of CPU time used during encryption and decryption over a specific time interval. The CPU usage depends on the algorithm, data size, hardware capabilities, and implementation. It is as shown in Equations (3) and (4) below.

(3) $$U_{enc}=\left(\frac{T_{enc\_cpu}}{T_{enc\_wall}\times C}\right)\times 100$$

(4) $$U_{dec}=\left(\frac{T_{dec\_cpu}}{T_{dec\_wall}\times C}\right)\times 100$$

where

$U_{enc}=\;$CPU Usage during encryption (%)

$U_{dec}=\;$CPU Usage during decryption (%)

$T_{enc\_cpu}=$ total CPU time consumed for encryption

$T_{dec\_cpu}=\;$total CPU time consumed for decryption

$T_{enc\_wall}\;$ and $T_{dec\_wall}\;$= wall clock time taken

C = number of CPU cores

Memory usage refers to the amount of RAM (Random Access Memory) consumed by a system or application during the encryption or decryption process. It is measured as a percentage of memory usage during encryption and decryption consumed by an algorithm while processing data securely, as shown in Equations (5) and (6):

(5) $$U_{mem\_enc}=\;\left(\frac{M_{enc}}{M_{total}}\right)\;\times \;100$$

(6) $$U_{mem\_dec}=\left(\frac{M_{dec}}{M_{total}}\right)\;\times \;100$$

where

$U_{mem\_enc}$ = % of RAM used for encryption

$U_{mem\_dec}$ = % of RAM used for encryption

$M_{enc}$ = Memory used during encryption (MB)

$M_{dec}$ = Memory used during decryption (MB)

$M_{total}$ = Total available RAM on the system (MB)

### 5.2. Scalability Evaluation

The scalability analysis was guided directly by the constraints typical in edge computing environments used in IoT-based smart healthcare settings. Unlike cloud servers, edge devices operate with limited CPU capacity, restricted memory, and strict real-time response requirements. Because of these limitations, scalability cannot be evaluated only through encryption and decryption time. It must also account for how well an algorithm performs as data volume grows while running on resource-constrained hardware.

For this reason, three quantitative metrics were selected. Processing time captures how quickly the device can complete encryption and decryption as file sizes increase. CPU usage reflects the computational load placed on the edge node, which is important because edge processors typically have low clock speeds and cannot support heavy workloads for extended periods. Memory usage indicates how efficiently the algorithm uses available RAM, an essential factor for continuous patient monitoring, where multiple processes often run in parallel. The experimental design therefore increases file sizes from 256 KB up to 151 MB to simulate real-world scenarios where patient records accumulate and change frequently. By observing how each hybrid encryption scheme behaves under these growing data conditions, the analysis demonstrates not only performance but also the practical capacity of the edge device to handle future data expansion without system slowdown or failure.

## 6. Results and Discussion

### 6.1. Edge-Enabled Hybrid Encryption Performance Analysis

The focus of this study is to examine the durations required for encryption and decryption, the extent of CPU usage during these operations, and the amount of memory utilized by each technique. This extensive research highlights the relative efficiency of several encryption techniques in real-life situations, offering significant perspectives on their practicality and resource utilization.

#### 6.1.1. Encryption and Decryption Time

When utilizing the ECC + HMAC-SHA256 + Blowfish encryption algorithm, a pattern of longer encryption times corresponds to bigger file sizes. Meanwhile, the ECC + HMAC-SHA256 + Chacha20 algorithm’s encryption time for various file sizes has a tendency toward longer encryption times as file size increases. The encryption time increases noticeably to 2.033 s as the file size increases, especially in the case of the highest file size of 151 MB. Therefore, these correlations emphasize the impact of file size on the performance of the algorithm, with larger files requiring more processing time for encryption.

Based on the performance, the ECC + HMAC-SHA256 + AES approach outperforms the other options. Without the rapid increase observed with Salsa20 and Chacha20, the approach under consideration has stable and predictable encryption time for larger file sizes. Blowfish has a linear link between encryption time and file size, making it an option, though it is less efficient than AES. The Salsa20 method’s quick increase in encryption time as file size increases suggests it may not be ideal for large datasets.

**Figure 3:**
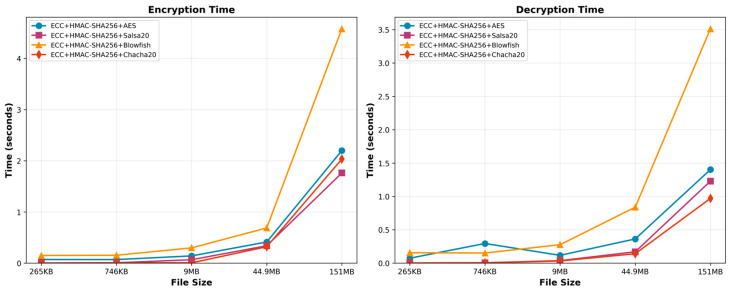
Time performance evaluation.

The time evaluation suggests that the best algorithm depends on the application context and file size (Figure 3). ECC + HMAC-SHA256 + Salsa20 Encryption is best for quick file size decryption. ECC + HMAC-SHA256 + AES Encryption can also be used to prioritize smaller files. Due to their slower decryption speeds, Blowfish and Chacha20 may perform poorly on larger data. Therefore, when choosing an algorithm for an application, it is vital to consider the compromises between encryption level and decryption efficiency.

#### 6.1.2. CPU Usage

**Figure 4:**
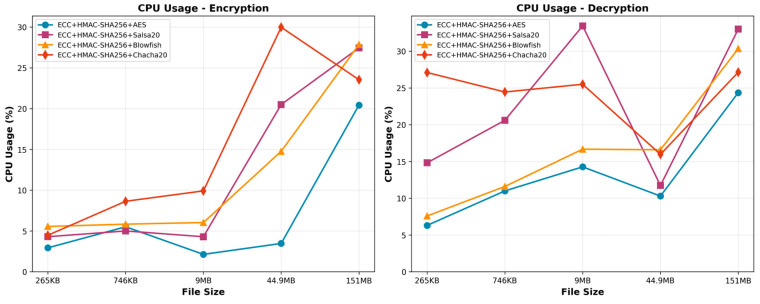
CPU usage of the encryption and decryption.

Based on CPU evaluation, the study showed that Salsa20 is the best CPU algorithm for varied file sizes (Figure 4). AES offers robust encryption but increases CPU consumption as file size increases. AES uses more system resources than Salsa20, hence it may not be as efficient at managing larger files. The ECC + HMAC-SHA256 + AES method is the preferred choice for decryption processing in terms of CPU efficiency. Salsa20’s architecture makes it ideal for resource-constrained embedded devices. It uses 27.44% CPU for the largest file (151 MB) and regularly uses low CPU. This shows that it can process data efficiently without straining the CPU. As file size increases, Salsa20’s CPU utilization scales effectively. The combination of ECC, HMAC-SHA256, and AES offers robust encryption but increases CPU consumption as file size increases.

The ECC + HMAC-SHA256 + AES algorithm consistently exhibited better performance in file decryption than the other algorithms under evaluation. This remarkable advantage can be attributed to the algorithm’s efficient use of CPU usage. As the size of the file increased in size, the CPU usage for the ECC + HMAC-SHA256 + AES algorithm likewise increased, although to a smaller degree when compared to the other techniques.

The utilization of ECC + HMAC-SHA256 AES demonstrated enhanced efficiency in managing this increased load, leading to reduced CPU usage. On the other hand, it was observed that ECC + HMAC-SHA256 + Salsa20, ECC + HMAC-SHA256 + Blowfish, and ECC + HMAC-SHA256 + Chacha20 demonstrated a rise in CPU usage with increasing file size, suggesting an inefficient utilization of CPU resources. Consequently, taking into account all of the above, it can be concluded that the ECC + HMAC-SHA256 + AES method is the preferred choice for decryption processes in terms of CPU efficiency.

#### 6.1.3. Memory Usage

**Figure 5:**
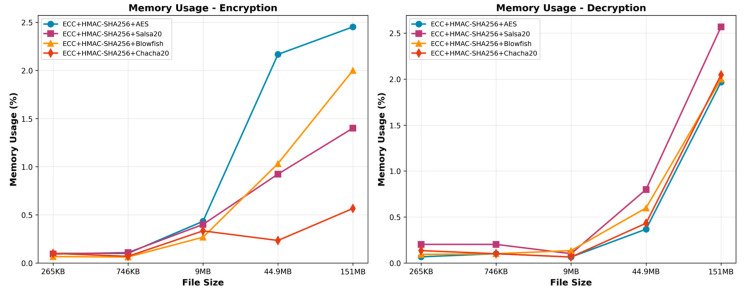
Memory usage of the encryption and decription.

In the context of memory usage, the combination of ECC, HMAC-SHA256, and Salsa20 performs best when considering different file sizes (Figure 5). The memory usage remains continuously low throughout various file sizes. This combination consistently utilizes the minimum amount of memory, while ensuring a predictable scaling pattern. This characteristic makes it the preferred option in scenarios when the encryption process requires minimal memory use. The algorithm comprising ECC, HMAC-SHA256, and AES emerges as the most optimal solution for decryption due to its consistent and efficient utilization of memory resources, rendering it a dependable option for processing data of various sizes.

#### 6.1.4. Analysis

The experiment facilitates code portability and reduces debugging overhead during edge deployment. This choice enables valid comparative analysis of algorithmic efficiency, scalability, and resource consumption under ideal conditions, providing a reliable baseline for future adaptation to lower-powered edge hardware. The results thus serve as a reference point for evaluating trade-offs and guiding implementation decisions in real-world healthcare deployments.

Based on these aspects, the edge-enabled hybrid approach assesses the processing time required, the usage of CPU during these tasks, and memory usage required, for both encryption and decryption. The performance evaluation of encryption algorithms has a significant implication for future implementation in real-world healthcare systems. The results emphasize the importance of selecting a suitable encryption technique based on the overall healthcare system requirements, including different types of data, and its characteristics. For instance, ECC + HMAC-256 + AES demonstrates consistent performance across various file sizes; therefore, it is suitable for systems that handle different medical data types. On the other hand, ECC + HMAC-SHA256 + Salsa20 achieves faster results when used with smaller file sizes. The study also underscores the critical nature of resource utilization, with Salsa20 showing efficient CPU usage across different file sizes, while AES exhibits effective memory usage during decryption. ChaCha20’s ARX-based design provides exceptional performance for small-to-medium healthcare records (≤10 MB), with minimal memory overhead ideal for edge devices. However, the lack of hardware acceleration on commodity processors results in linear scaling challenges for large datasets. The algorithm’s constant-time execution and absence of lookup tables offer superior resistance to cache-timing attacks—a critical consideration for distributed healthcare environments, where adversaries may have physical access to edge nodes.

The findings highlight that a conventional approach is not sufficient anymore, while there is a need for a balanced approach, considering security, efficiency, and resource constraints when implementing encryption in healthcare systems. Performance obtained from the algorithms with different file sizes also shows that HIE systems might benefit from employing multiple encryption methods, as a single and conventional is not appropriate when it comes to specific data types and security requirements for each transaction. This research contributes to the development of more secure, efficient, and responsive healthcare data sharing systems, potentially improving the overall quality and timeliness of patient care.

### 6.2. Scalability Assessment

Scalability assessment was performed through a comprehensive experimental setup that simulated varying data sizes to evaluate system performance under dynamic conditions. This assessment is important in IoT-based smart healthcare environments, particularly those operating within edge computing infrastructures, where patient data is collected, growing, and changing between parties involved. Patient data was generated with four distinct file sizes ranging from 256 KB (4500 records) to 151 MB (2,340,689 records). Each dataset undergoes a constant process of key exchange, hashing, encryption, and decryption for different encryption algorithms. This incremental increase in file size does not allow for an observation of how each algorithm handles increasing data volumes.

The results show that the system has the ability to maintain operational integrity and acceptable performance levels while the file size increases, unlike most of the existing approaches in the literature, which disregard the scalability aspect when discussing secure HIE systems. By incorporating this evaluation, the study provides valuable insights into how well encryption algorithms are able to perform under various file sizes, which is a critical requirement for real-world healthcare scenarios. Overall, the findings confirm that the proposed edge-enabled hybrid approach is scalable and capable of supporting secure HIE, even as the amount of data grows significantly, which is a normal situation in this domain. This ensures that the system remains robust and responsive in high-volume environments typical of smart healthcare infrastructures.

## 7. Conclusions and Future Work

This study introduced an edge-enabled hybrid encryption framework tailored for secure and efficient health information exchange (HIE) in IoT-based smart healthcare systems. By integrating ECC for secure key exchange, HMAC-SHA256 for integrity verification, and multiple symmetric algorithms for confidentiality, the proposed architecture demonstrated promising performance across encryption time, CPU usage, memory consumption, and scalability.

To ensure practical deployment, future work will focus on integrating the framework into real-world HIE workflows such as HL7 FHIR systems, while benchmarking across heterogeneous edge devices like Raspberry Pi, Jetson Nano, and ARM Cortex-M. This will enable a granular evaluation of performance variability and help identify optimal cryptographic configurations tailored to device-specific capabilities. Optimization strategies including lightweight cryptographic tuning, parallel processing, and memory reduction could also be explored to enhance responsiveness and energy efficiency. Additionally, dynamic algorithm-selection mechanisms and adversarial resilience testing could potentially be developed to support context-aware encryption. This may involve embedding lightweight rule-based decision engines or adaptive models at the edge to autonomously select the most efficient and secure encryption pathway. Furthermore, security validation under adversarial conditions—including replay, man-in-the-middle, and side-channel attacks—will be conducted to ensure robustness and resilience.

Thus, the demonstrated efficiency of the ECC + HMAC-SHA256 + AES hybrid approach underscores the importance of edge-enabled encryption for securing health data during transmission and storage in IoT-based smart healthcare systems. However, further research is needed to explore alternative cryptographic combinations, device capabilities, and dynamic selection methods that adapt to varying data attributes and operational contexts. These efforts aim to advance secure, scalable, and regulation-aligned encryption solutions for next-generation healthcare information exchange in IoT-based smart healthcare systems.

## Figures and Tables

**Table 1:** Comparison of existing techniques and the performance metrics used.

Authors	Proposed Techniques	Performance Metrics			Scalability Evaluation
		Encryption/Decryption Time	Memory Usage	CPU Usage	
Khan et al. [9]	Data Substitution–Caesar Cipher + Improved IECC	/	X	X	X
Qiu et al. [17]	AES Encryption	X	/	/	X
Xiong et al. [15]	Ciphertext-Policy Attribute-Based Encryption (CP-ABE)	/	/	/	/
Nagarajan et al. [13]	RES-256 Algorithm	X	X	X	X
Abhishek et al. [16]	Updated Blowfish Algorithm	/	/	/	X
Kaliyaperumal & Sammy [10]	Attribute-Based Encryption (ABE)	X	X	X	/
Olusogo Popoola et al. [11]	Hybrid ECC-256r1 + AES-128 (EAX Mode)	/	/	/	X
Younes Lahraoui et al. [12]	Hash-based ECC with ECDH Protocol	X	X	X	X

**Table 2:** Comparative summary of existing works vs. proposed framework.

Study	Encryption Scheme	Edge Optimization	HIE Workflow Integration	Performance Metrics Evaluated
Khan et al. [9]	Caesar Cipher + Improved IECC	X	X	Memory, CPU
Popoola et al. [11]	ECC-256R1 + AES-128 (EAX Mode)	X	✓	Encryption time
Lahraoui et al. [12]	Hash-based ECC + ECDH	X	X	Encryption time, CPU, memory
Proposed Framework	ECC + HMAC-SHA256 + AES/ChaCha20/etc.	✓	✓	Encryption time, CPU, memory, scalability

**Table 3:** File encryption and decryption time (seconds).

Hybrid Encryption and File Size	Algorithm 1		Algorithm 2		Algorithm 3		Algorithm 4	
	Encryption	Decryption	Encryption	Decryption	Encryption	Decryption	Encryption	Decryption
265 KB (num_record = 4500)	0.07103	0.07402	0.00299	0.00252	0.14954	0.15585	0.00319	0.00189
746 KB (num_record = 12,500)	0.06974	0.29430	0.00704	0.00547	0.15379	0.15140	0.00620	0.00387
9 MB (num_record = 145,000)	0.14091	0.11680	0.06661	0.03732	0.29777	0.27650	0.00580	0.03212
44.9 MB (num_record = 710,500)	0.41276	0.36250	0.33931	0.17004	0.68711	0.83898	0.32076	0.14087
151 MB (num_record = 2,340,689)	2.19853	1.40220	1.760908	1.22898	4.57712	3.51267	2.03300	0.97007

**Table 4:** CPU usage for encryption and decryption (%).

Hybrid Encryption and File Size	ECC + HMAC-SHA256 + AES		ECC + HMAC-SHA256 + Salsa20		ECC + HMAC-SHA256 + Blowfish		ECC + HMAC-SHA256 + Chacha20	
	Encryption	Decryption	Encryption	Decryption	Encryption	Decryption	Encryption	Decryption
265 KB (num_record = 4500)	2.93348	6.30372	4.30022	14.81733	5.55283	7.58503	4.47473	27.10007
746 KB (num_record = 12,500)	5.51403	10.99371	5.00106	20.59971	5.83686	11.59997	8.64105	24.45003
9 MB (num_record = 145,000)	2.13341	14.27000	4.29551	33.44332	6.03333	16.67337	9.91673	25.50367
44.9 MB (num_record = 710,500)	3.47041	10.29334	20.50342	11.73335	14.76573	16.59371	29.97481	15.96678
151 MB (num_record = 2,340,689)	20.43368	24.36334	27.44083	33.00006	27.85906	30.33997	23.54103	27.14289

**Table 5:** Memory usage for encryption and decryption process (%).

Hybrid Encryption and File Size	ECC + HMAC-SHA256 + AES		ECC + HMAC-SHA256 + Salsa20		ECC + HMAC-SHA256 + Blowfish		ECC + HMAC-SHA256 + Chacha20	
	Encryption	Decryption	Encryption	Decryption	Encryption	Decryption	Encryption	Decryption
265 KB (num_record = 4500)	0.10042	0.06761	0.09342	0.20284	0.06694	0.09342	0.10042	0.13522
746 KB (num_record = 12,500)	0.10042	0.10142	0.10853	0.20284	0.06258	0.10142	0.06965	0.10142
9 MB (num_record = 145,000)	0.43324	0.06525	0.40052	0.10142	0.26808	0.13522	0.33399	0.06524
44.9 MB (num_record = 710,500)	2.16667	0.36799	0.92342	0.79972	1.03233	0.59901	0.23428	0.43262
151 MB (num_record = 2,340,689)	2.45227	1.96709	1.40076	2.56718	2.00047	2.00047	0.56653	2.04644

## Data Availability

No real-world data were used in this study. The dataset used was synthetically generated using a Python-based simulation model that reproduces statistical characteristics of real-world healthcare data.

## References

[1] Butt S.A., Diaz-Martinez J.L., Jamal T., Ali A., De-La-Hoz-Franco E., Shoaib M. (2019). IoT Smart Health Security Threats. Proceedings of the 2019 19th International Conference on Computational Science and Its Applications (ICCSA).

[2] Ghamari M., Janko B., Sherratt R.S., Harwin W., Piechockic R., Soltanpur C. (2016). A Survey on Wireless Body Area Networks for Ehealthcare Systems in Residential Environments. Sensors.

[3] Manogaran G., Lopez D., Thota C., Abbas K.M., Pyne S., Sundarasekar R., Qudrat-Ullah H., Tsasis P. (2017). Big Data Analytics in Healthcare Internet of Things. Understanding Complex Systems.

[4] Esmaeilzadeh P. (2020). The Effect of the Privacy Policy of Health Information Exchange (HIE) on Patients’ Information Disclosure Intention. Comput. Secur..

[5] Bashir A., Mir A.H. (2019). Secure Framework for Internet of Things Based E-Health System. Int. J. E-Health Med. Commun..

[6] Burhan M., Rehman R.A., Khan B., Kim B.S. (2018). IoT Elements, Layered Architectures and Security Issues: A Comprehensive Survey. Sensors.

[7] Nidhya R., Kumar M., Maheswar R., Pavithra D., Rani S., Rajagopal M., Kumar N., Shah S.H.A. (2022). Security and Privacy Issues in Smart Healthcare System Using Internet of Things. IoT-Enabled Smart Healthcare Systems, Services and Applications.

[8] Tang M., Zeng L., Zeng Z., Liu J., Yuan J., Wu D., Lu Y., Zi J., Ye M. (2021). Proteomics Study of Colorectal Cancer and Adenomatous Polyps Identifies TFR1, SAHH, and HV307 as Potential Biomarkers for Screening. J. Proteom..

[9] Khan M.A., Quasim M.T., Alghamdi N.S., Khan M.Y. (2020). A Secure Framework for Authentication and Encryption Using Improved ECC for IoT-Based Medical Sensor Data. IEEE Access.

[10] Kaliyaperumal K., Sammy F. (2022). An Efficient Key Generation Scheme for Secure Sharing of Patients Health Records Using Attribute Based Encryption. Proceedings of the 2022 International Conference on Communication, Computing and Internet of Things, IC3IoT 2022—Proceedings.

[11] Popoola O., Rodrigues M.A., Marchang J., Shenfield A., Ikpehai A., Popoola J. (2024). An Optimized Hybrid Encryption Framework for Smart Home Healthcare: Ensuring Data Confidentiality and Security. Internet Things.

[12] Lahraoui Y., Lazaar S., Amal Y., Nitaj A. (2024). Securing Data Exchange with Elliptic Curve Cryptography: A Novel Hash-Based Method for Message Mapping and Integrity Assurance. Cryptography.

[13] Nagarajan S.M., Deverajan G.G., Kumaran U., Thirunavukkarasan M., Alshehri M.D., Alkhalaf S. (2022). Secure Data Transmission in Internet of Medical Things Using RES-256 Algorithm. IEEE Trans. Ind. Inform..

[14] Qiu H., Qiu M., Liu M., Memmi G. (2020). Secure Health Data Sharing for Medical Cyber-Physical Systems for the Healthcare 4.0. IEEE J. Biomed. Health Inform..

[15] Xiong S., Ni Q., Wang L., Wang Q. (2020). SEM-ACSIT: Secure and Efficient Multiauthority Access Control for IoT Cloud Storage. IEEE Internet Things J..

[16] Abhishek B., Panjanathan R., Raja Sarobin V., Edwin Raja B., Narendra M. (2022). Data Security in E-Health Monitoring System. Mater. Today Proc..

[17] Qiu T., Chi J., Zhou X., Ning Z., Atiquzzaman M., Wu D.O. (2020). Edge Computing in Industrial Internet of Things: Architecture, Advances and Challenges. IEEE Commun. Surv. Tutor..

[18] Gowthamani R., Manoj S.O., Rani K.S.K. (2025). Empowering Integrity and Confidentiality in Smart Healthcare Systems through Effective Edge Cryptographic Strategies. Automatika.

[19] Singh A., Chatterjee K. (2023). Edge Computing Based Secure Health Monitoring Framework for Electronic Healthcare System. Clust. Comput..

[20] Parah S.A., Kaw J.A., Bellavista P., Loan N.A., Bhat G.M., Muhammad K., Albuquerque V.H.C. (2021). de Efficient Security and Authentication for Edge-Based Internet of Medical Things. IEEE Internet Things J..

[21] Baban A.B., Hameed S.A. (2023). Securing a Web-Based Hospital Management System Using a Combination of AES and HMAC. Iraqi J. Electr. Electron. Eng..

[22] Pronika, Tyagi S.S. (2021). Secure Data Storage in Cloud Using Encryption Algorithm. Proceedings of the 3rd International Conference on Intelligent Communication Technologies and Virtual Mobile Networks, ICICV 2021.

[23] Barker E. (2016). Recommendation for Key Management—Part 1: General.

[24] Grignani W., Santana K.G.Q., Santos D.A., Dilillo L., Melo D.R. Implementation and Reliability Evaluation of a ChaCha20 Stream Cipher Hardware Accelerator. Proceedings of the 2024 IEEE International Symposium on Defect and Fault Tolerance in VLSI and Nanotechnology Systems (DFT).

[25] Palka P., Perez R.A., Fang T., Saniie J. Design Flow of Blowfish Symmetric-Key Block Cipher on FPGA. Proceedings of the 2022 IEEE International Conference on Electro Information Technology (eIT).

[26] Le V.T.D., Pham H.L., Duong T.S., Tran T.H., Nguyen Q.D.N., Nakashima Y. RHCP: A Reconfigurable High-Efficient Cryptographic Processor for Decentralized IoT Platforms. Proceedings of the 2023 15th International Conference on Knowledge and Systems Engineering (KSE).

